# To Hasten the Action of Quinine

**Published:** 1883-05

**Authors:** 


TO HASTEN THE ACTION OF QUININE.

Dr. Starhe, in *Berliner Klin. Wochenschrift* advises that before swallowing powder or pills of quinine, a weak tartaric acid lemonade be taken. This precedure not only greatly accelerates the solution and absorption of quinine, rendering its physiological action much more prompt, but also obviates that unpleasant gastric irritation so common after the administration of large doses of this drug.—*Medical and Surgical Reporter.*

